# Ligustilide Suppresses Macrophage-Mediated Intestinal Inflammation and Restores Gut Barrier via EGR1-ADAM17-TNF-α Pathway in Colitis Mice

**DOI:** 10.34133/research.0864

**Published:** 2025-09-02

**Authors:** Yanyang Li, Yequn Wu, Jing Liang, Peiqi Chen, Shihua Xu, Yumei Wang, Zhi Jiang, Xudong Zhu, Chaozhan Lin, Yang Yu, Hailin Tang

**Affiliations:** ^1^ School of Pharmaceutical Sciences, Guangzhou University of Chinese Medicine, Guangzhou 510006, China.; ^2^ Department of Perioperative Research Centre of Chinese Medicine, the Second Affiliated Hospital of Guangzhou University of Chinese Medicine, Guangzhou 510120, China.; ^3^ Markey Cancer Center, University of Kentucky, Lexington, KY 40536, USA.; ^4^ State Key Laboratory of Oncology in South China, Guangdong Provincial Clinical Research Center for Cancer, Sun Yat-sen University Cancer Center, Guangzhou 510060, China.

## Abstract

Ulcerative colitis is a chronic nonspecific intestinal inflammatory disease, which usually occurs in the rectal and colonic mucosa and submucosa. Ligustilide, a major component derived from *Angelica sinensis* (Oliv.) Diels, exerts anti-inflammation effect. However, its impact and molecular mechanism on colitis remain obscure. In this study, in vivo and in vitro experiments verified that ligustilide protected against colitis by suppressing macrophage-mediated inflammation and repairing intestinal barrier. Of note, we utilized a thermal proteome profiling strategy to preliminarily find early growth response factor 1 (EGR1) as a target of ligustilide. Cellular thermal shift assay, drug affinity responsive target stability, and surface plasmon resonance analysis revealed that ligustilide directly targeted His386 to bind to EGR1. Furthermore, RNA-sequencing, dual luciferase reporter gene assay, and rescue experiments illustrated that ligustilide disturbed the nuclear translocation of EGR1 and broke its combination with a disintegrin and metalloproteinase 17 (ADAM17) promoter, thereby inhibiting ADAM17 transcription and downstream tumor necrosis factor-α (TNF-α) production, as well as expression of inflammatory proteins cyclooxygenase 2 and inducible nitric oxide synthase. Finally, the in vivo experiment with EGR1 overexpression proved that EGR1 was essential for the protective effects of ligustilide on colitis mice. Taken together, our study demonstrates that ligustilide targets EGR1 to inhibit the EGR1-ADAM17-TNFα pathway, thus alleviating macrophage-mediated intestinal inflammation and restoring gut barrier.

## Introduction

Ulcerative colitis (UC) is characterized by chronic intestinal inflammation accompanied by clinical manifestations such as diarrhea, abdominal pain, and hematochezia [1]. Currently, the existing drugs for UC are non-steroidal anti-inflammatory drugs, glucocorticoids, and immunosuppressants, which might display apparent side effects, drug resistance, and high relapse rates after drug withdrawal [2]. For instance, mesalazine has adverse reactions such as diarrhea, drug-induced interstitial nephritis, and severe allergies [3,4]. Therefore, there is urgency to find effective and safe drugs for UC.

Early growth response factor 1 (EGR1) is a transcription factor involved in the processes of tissue damage, immune response, and fibrosis [5]. Studies have shown that EGR1 participated in inflammation and apoptosis by regulating downstream extracellular regulated protein kinases (ERK), c-Jun N-terminal Kinase (JNK), and p38 mitogen-activated protein kinase (MAPK) pathways [6,7]. In the lipopolysaccharide (LPS)-induced sepsis mouse model, inhibiting the expression of EGR1 reduced pro-inflammatory cytokines production to mitigate tissue damage and increase the survival rate of mice [6]. Besides, it is found that EGR1 expression in the UC patient is significantly increased compared with the healthy volunteers [1]. A dramatic activation of EGR1 signaling was observed in intestinal epithelial cells stimulated by LPS or intestinal flora of UC patients [2,8].

Ligustilide, a type of phthalide derivative abundant in *Angelica sinensis* and *Ligusticum sinense*, exhibits extensive biological activities, including anti-inflammatory, anti-cancer, and neuroprotective activities. Ligustilide regulated inflammation, cellular oxidative damage, and fibrosis through the AMPK/GSK-3β/Nrf2 pathway, thereby alleviating glucolipotoxicity-incurred cardiomyocyte dysfunction [9]. In atopic dermatitis models, ligustilide up-regulates filaggrin and SPTLC1, and reduces tumor necrosis factor-α (TNF-α), interferon-γ, and interleukin-6 (IL-6) to reinforce skin barrier function [10]. Our previous study illustrated that Angelica oil suppressed the S100A8/A9 signaling pathway to restore gut barrier function in dextran sulfate sodium (DSS)-induced colitis mice and the primary active ingredient is ligustilide [11]. Huang et al. [12] also reported that ligustilide attenuates experimental colitis mice by reducing the production of pro-inflammatory cytokines. Nevertheless, the effect and mechanism of ligustilide on colitis mice remain obscure.

In this study, the in vivo anti-colitis effect and in vitro anti-inflammatory impact of ligustilide were confirmed by the establishment of the DSS-induced UC mouse model. We determined that ligustilide could target EGR1 and limit its transcriptional activities. A mechanism study uncovered that ligustilide disrupted the interaction of EGR1 with a disintegrin and metalloproteinase 17 (ADAM17) promoter, hampering TNF-α production and expressions of inflammatory-related proteins cyclooxygenase 2 (COX-2) and inducible nitric oxide synthase (iNOS). Additionally, the EGR1 overexpression (OE-EGR1) mouse model ascertained the fundamental role of EGR1 in ligustilide exerting protective effect against colitis. To sum up, our findings provide an EGR1-targeting natural compound, ligustilide, which might become a candidate for the treatment of UC.

## Results

### Ligustilide improved colitis-associated symptoms in mice

**Fig. 1.**
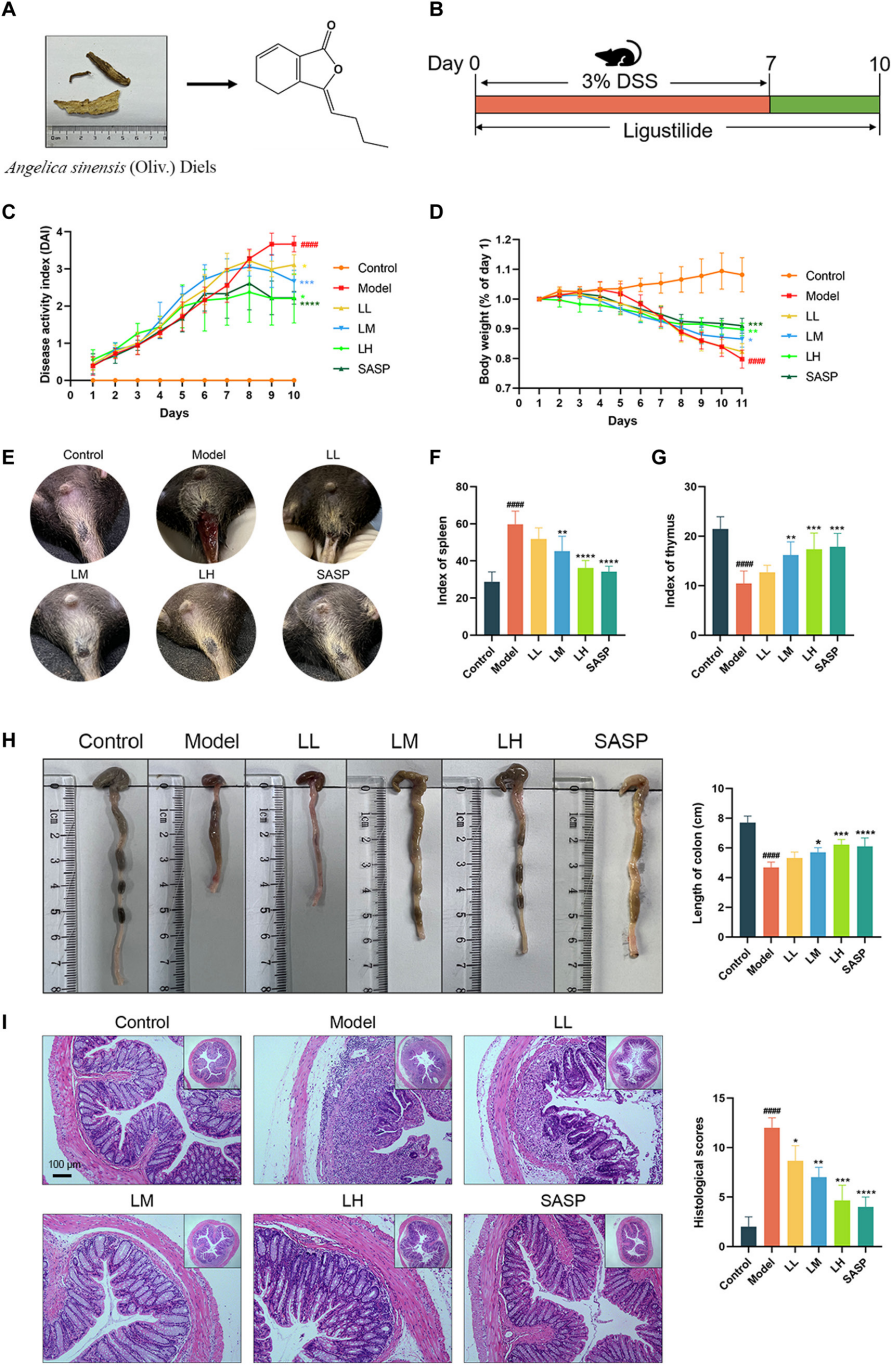
Ligustilide improved colitis-associated symptoms in mice. (A) The structure of ligustilide. (B) Establishment of the colitis model and administration of ligustilide. (C) DAI scores. (D) Weight changes of colitis mice. (E) Observation of anal bleeding in colitis mice. (F) Index of spleen. (G) Index of thymus. (H) Macroscopic observation of colon lengths. (I) H&E staining of colonic pathological structure. LL, low dose of ligustilide; LM, medium dose of ligustilide; LH, high dose of ligustilide. ^####^*P <* 0.0001 vs. control group. ^*^P < 0.05, ^**^P < 0.01, ^***^P < 0.001, ^****^P < 0.0001 vs. model group.

The structure of ligustilide is presented in Fig. 1A. As depicted in Fig. 1B, colitis mice were induced by 3% DSS for 7 days and administrated with ligustilide for 10 days. DSS-induced colitis mice showed significant weight loss, hematochezia, and anal bleeding. Compared with colitis mice, ligustilide treatment was beneficial to reducing weight loss, alleviating anal bleeding, and lowering the disease activity index (DAI) score (Fig. 1C to E). In addition, ligustilide could significantly increase the index of spleen, reduce the index of thymus, and restore the colon length of colitis mice (Fig. 1F to H). Besides, we observed that ligustilide could effectively improve the desquamation of colon epithelial cells, inflammatory cell infiltration, and muscle layer extensive edema (Fig. 1I). Therefore, our in vivo data confirmed the treatment effect of ligustilide on colitis mice.

### Ligustilide repaired intestinal mucosal barrier in colitis mice

**Fig. 2.**
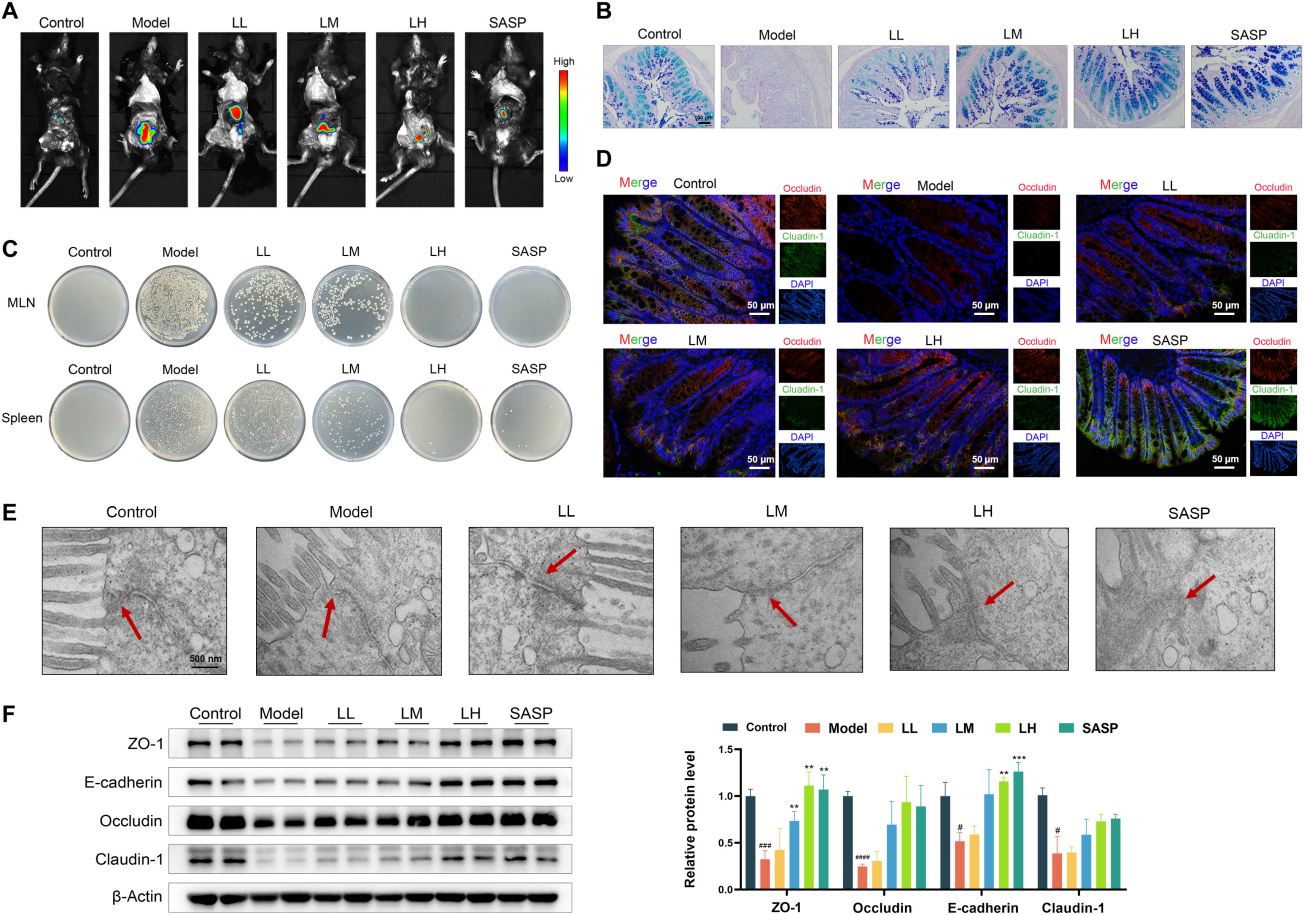
Ligustilide repairing intestinal mucosal barrier in colitis mice. (A) Small-animal imaging observed the leakage area of FD4. (B) AP-PAS staining of goblet cells. (C) The bacterial load of the spleen and MLNs. (D) Immunofluorescence observed ZO-1 and Claudin-1 expressions. (E) The ultrastructure of the colonic tight junction was observed by TEM. (F) ZO-1, Occludin, Claudin-1, and E-cadherin expression levels by Western blot. LL, low dose of ligustilide; LM, medium dose of ligustilide; LH, high dose of ligustilide. ^#^*P <* 0.05, ^###^*P <* 0.001, ^####^*P <* 0.0001 vs. control group. ^**^P < 0.01, ^***^P < 0.001 vs. model group.

Intestinal mucosal barrier, a crucial defense line, prevents against the occurrence and development of UC. Therefore, evaluating the integrity of intestinal barrier can effectively reflect the UC therapeutic effect of ligustilide. Compared with the control group, the leakage area of 4 kDa fluorescein isothiocyanate dextran (FD4) was enlarged, the number of colonic goblet cells was decreased, and a large number of bacteria from their spleens and mesenteric lymph nodes (MLNs) were cultured in colitis mice. Ligustilide could considerably reduce the leakage area of FD4 and increase goblet cell number and the bacterial load of the spleen and MLNs (Fig. 2A to C). Besides, the ultrastructure of the colon epithelium tight junctions was observed by transmission electron microscopy (TEM). Tight junction is an important indicator for maintaining the integrity of the intestinal barrier. The results of TEM showed that the tight junction structure of colitis mice was damaged, while ligustilide could effectively restore the tight junction structure (Fig. 2E). Concurrently, it was observed that ligustilide notably increased expressions of ZO-1, Occludin, Claudin-1, and E-cadherin (Fig. 2D and F). In conclusion, ligustilide can reduce gut permeability and repair intestinal mucosal barrier in colitis mice.

### Ligustilide alleviated macrophage-mediated intestinal inflammation

**Fig. 3.**
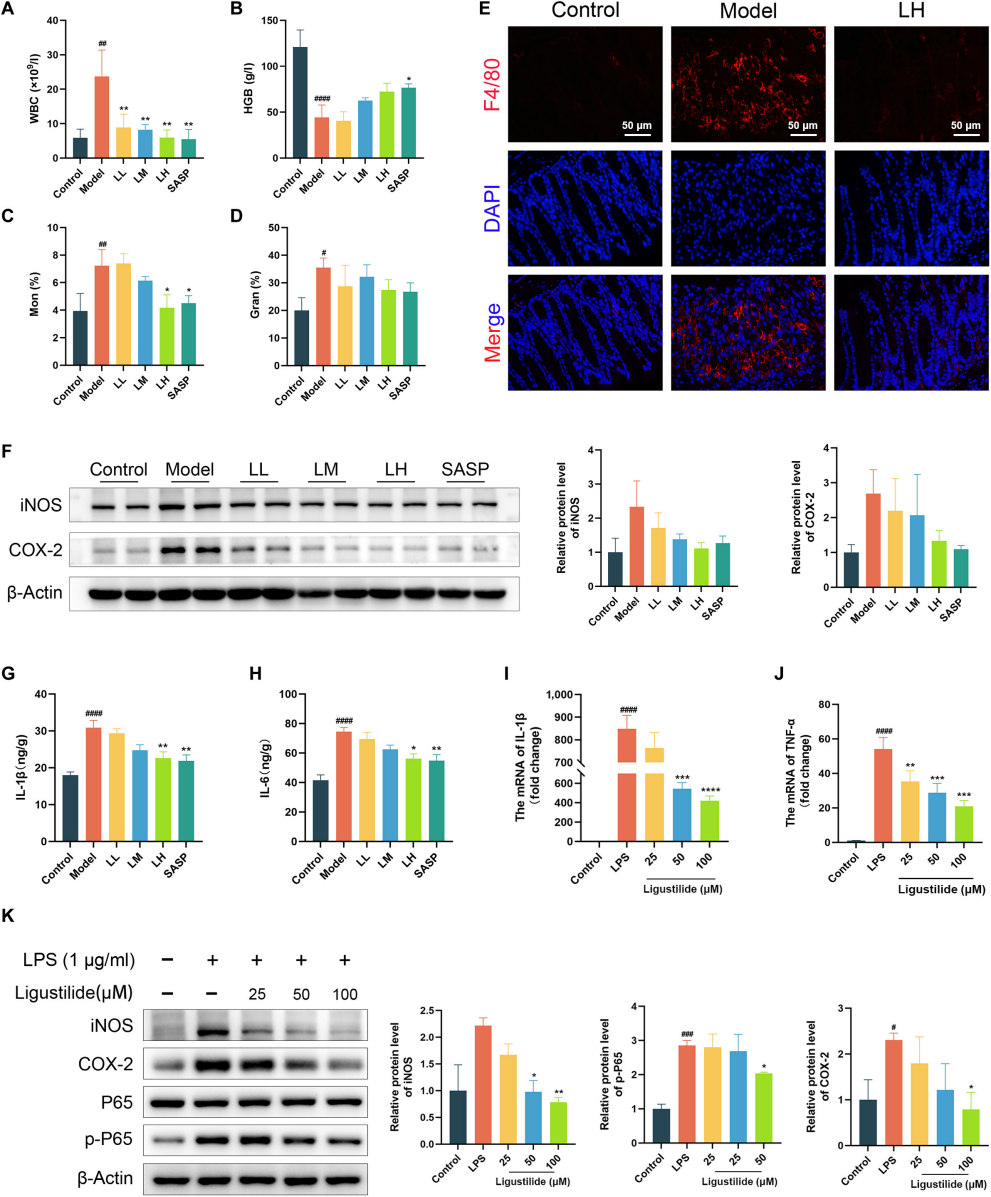
Ligustilide alleviates macrophage-mediated intestinal inflammation. (A) The number of white blood cells. (B) The content of hemoglobin. (C) The ratio of monocytes. (D) The ratio of granulocytes. (E) Immunofluorescence observed macrophage infiltration in colon. (F) Western blot detected iNOS and COX-2 expressions. (G and H) The content of IL-1β and IL-6 of colon tissues. (I and J) IL-1β and TNF-α mRNA level of RAW264.7 cell. (K) Western blot detected iNOS and COX-2 expressions of RAW264.7 cell. LL, low dose of ligustilide; LM, medium dose of ligustilide; LH, high dose of ligustilide. ^#^*P <* 0.05, ^##^*P <* 0.01, ^###^*P <* 0.001, ^####^*P <* 0.0001 vs. control group. ^*^P < 0.05, ^**^P < 0.01, ^***^P < 0.001, ^****^P < 0.0001 vs. model group or LPS group.

Inflammation is the key pathogenic basis of UC. Under the inflammatory microenvironment, various pathogenic processes such as the intestinal mucosal barrier, immune response, epithelial–mesenchymal transition, and fibrosis change and jointly participate in the progression of UC [13–15]. Accordingly, we assessed intestinal inflammation in colitis mice treated with ligustilide. We found that ligustilide could substantially reduce the number and proportion of white blood cells and monocytes, increase hemoglobin content (Fig. 3A to D), and reduce F4/80+ macrophage infiltration (Fig. 3E) and expressions of inflammatory proteins iNOS and COX-2 (Fig. 3F). Besides, ligustilide considerably lowered secretion of inflammatory factors IL-1β and IL-6 in the colon tissue (Fig. 3G and H).

To further verify the effect of ligustilide on macrophage inflammation, we selected the RAW264.7 cell line for further experiments. Ligustilide with different concentrations (range from 0 to 200 μM) displayed non-toxic effect on RAW264.7 cells (Fig. S1). After being stimulated by LPS, the morphology of RAW264.7 cells changed from round to long spindle-shaped, which was restored by treating with ligustilide (Fig. S2). Besides, we observed that ligustilide could significantly reduce the mRNA levels of IL-1β and TNF-α (Fig. 3I and J), as well as the expression of inflammatory proteins including iNOS, COX-2, and p-P65/P65 (Fig. 3K). Consequently, in vivo and in vitro results demonstrated the inhibitory effect of ligustilide on macrophage-mediated intestinal inflammation.

### Ligustilide targeted EGR1 and stabilized its structure by binding to His386

**Fig. 4.**
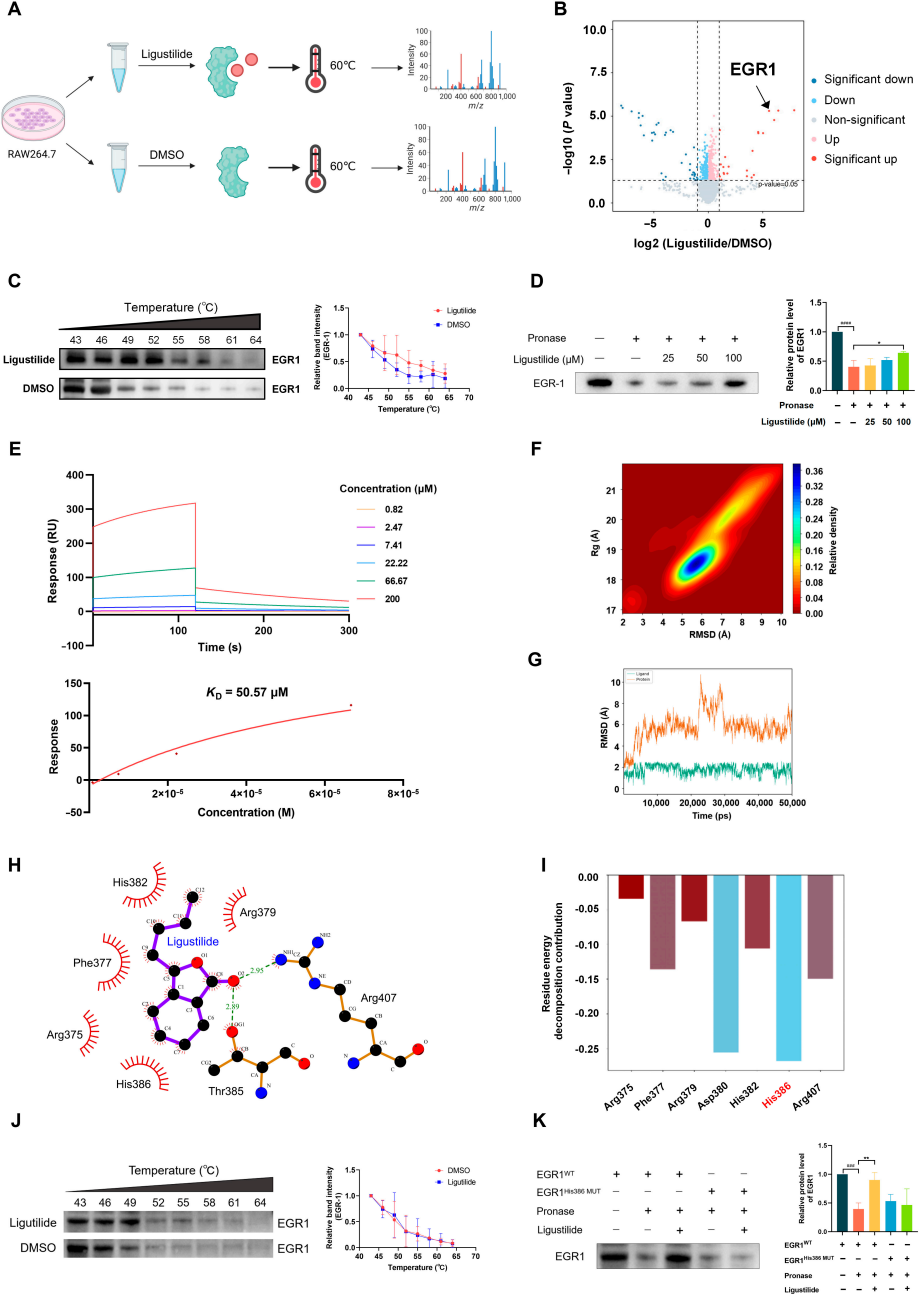
Ligustilide targeted EGR1 and stabilized its structure by binding to His386. (A) TPP workflow for target identification of ligustilide in RAW264.7 cell. (B) Ligustilide-interacting proteins were displayed by the volcano plot. (C) CETSA assay. (D) DARTS assay. (E) SPR analysis. (F) Gibbs free energy landscape of the ligustilide–EGR1 complex. (G) The RMSD of ligand ligustilide and EGR1 protein. (H) Molecule docking model of the ligustilide–EGR1 complex. (I) The residue energy decomposition of 7 binding pockets of EGR1 with ligustilide. (J) CETSA assay evaluated the interaction of EGR1 His386 ^MUT^ with ligustilide. (K) DARTS assay evaluated the interaction of EGR1 His386 ^MUT^ with ligustilide. ^###^*P*<0.001, ^####^*P <* 0.0001 vs. control group. ^*^P < 0.05, ^**^P < 0.01 vs. Pronase group.

To investigate the molecules targeted by ligustilide to inhibit macrophage inflammation, we used a thermal proteome profiling (TPP) strategy (Fig. 4A). As shown in Fig. 4B, we identified 8,034 proteins, out of which 345 proteins displayed high confidence (ligustilide/dimethyl sulfoxide [DMSO] ratio > 1; *P* value < 0.05). Combining the high thermal stability of the target molecule and current literatures on the pathogenesis of UC [16], we focused on EGR1 (Fig. 4B). To further gain the direct interaction strength between ligustilide and EGR1, we adopted cellular thermal shift assay (CETSA), drug affinity responsive target stability (DARTS), and surface plasmon resonance (SPR) techniques for verification. CETSA results showed that EGR1 protein gradually degraded at gradient-increasing temperatures, while ligustilide could delay EGR1 degradation and enhance its stability (Fig. 4C). DARTS results manifested that ligustilide dose-dependently prevented degradation of EGR1 by pronase (Fig. 4D). Consistently, the SPR study also confirmed ligustilide’s dose-dependent binding (from 0.82 to 200 μM) and the affinity constant *K*_D_ was 50.57 μM (Fig. 4E). The above results evidenced that ligustilide targeted EGR1.

To further explore the binding domain of EGR1 with ligustilide, molecular docking and molecular dynamics simulation were conducted. Dynamic visualization analysis of the binding trajectory observed that ligustilide could stably bind to EGR1 (Fig. S3). Gibbs free energy landscape map showed that the free energy of the ligustilide–EGR1 complex was lower when root-mean-square deviation (RMSD) ranged from 5 to 7 Å and Gg ranged from 18 to 19 Å (Fig. 4F and Fig. S4A). The RMSD and change of hydrogen bond formed by the ligustilide–EGR1 complex was stabilized from 30 to 50 ns (Fig. 4G and Fig. S4B). Solvent-accessible surface area analysis showed that the relative exposure area of ligustilide in the solvent ranged from 100 to 250 Å^2^, indicating that EGR1 tightly wrapped ligustilide (Fig. S4C). Molecular docking and molecular dynamics simulation predicted that there was interaction between ligustilide and His382, Arg379, Arg407, Thr385, His386, Arg375, and Phe377 of EGR1, of which the residue energy decomposition of His386 was the highest (Fig. 4H and I). As an additional proof of the binding between ligustilide and His386 of EGR1, EGR1^WT^ and EGR1 His386 ^MUT^ plasmids were constructed and transferred to HEK293T cells. The binding capacity between EGR1 His386 MUT and ligustilide was assessed by DARTS and CETSA. CETSA results indicated that ligustilide failed to offset the degradation of EGR1 within the temperature range of 43 to 64 °C in EGR1 His386 ^MUT^ HEK293T cells (Fig. 4J). The DARTS study showed that compared with EGR1 ^WT^ HEK293T cells, there was an obvious decline in the EGR1 expression level of EGR1 His386 ^MUT^ HEK293T cells treated with ligustilide (Fig. 4K), suggesting that His386 of EGR1 was critical for the binding of ligustilide. These observations demonstrated that ligustilide could directly bind to EGR1 by targeting His386.

### Ligustilide reduced inflammation response in macrophage by limiting the nuclear translocation of EGR1

**Fig. 5.**
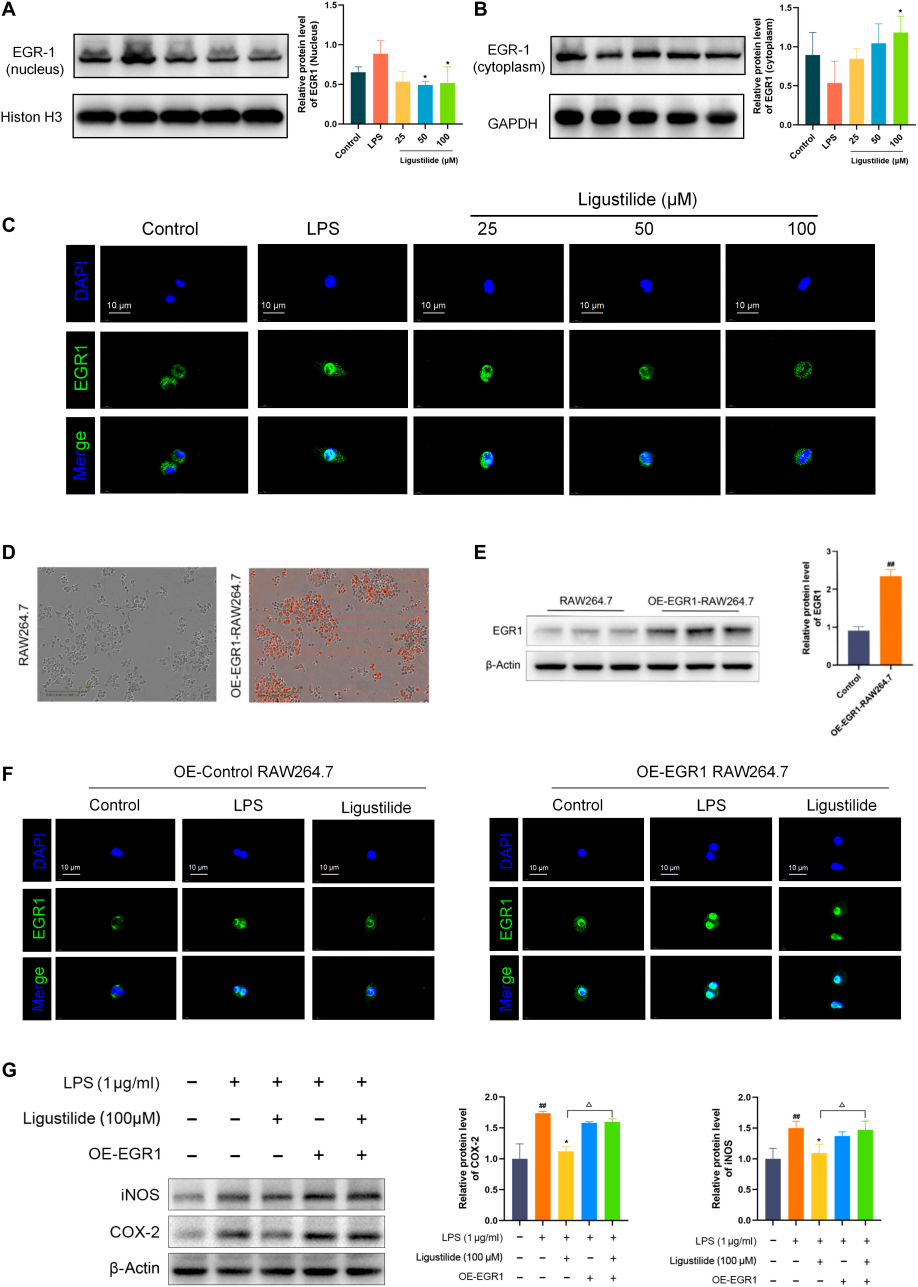
Ligustilide reduced inflammation response in macrophage by limiting the nuclear translocation of EGR1. (A) EGR1 protein expression in nucleus. (B) EGR1 protein expression in cytoplasm. (C) Immunofluorescence observed nuclear translocation of EGR1. (D) Transfection efficiency of EGR1 overexpression lentivirus. (E) EGR1 expression in RAW264.7 and OE-EGR1 RAW264.7 by Western blot. (F) Immunofluorescence assay evaluated whether EGR1 overexpression affected the nuclear translocation of EGR1. (G) Western blot assay evaluated whether EGR1 overexpression affected expressions of COX-2 and iNOS. OE-EGR1, EGR1 overexpression. ^#^*P <* 0.05, ^##^*P <* 0.01 vs. control group. ^*^P < 0.05, ^**^P < 0.01 vs. LPS group. ^△^ *P <* 0.05 vs. LPS + Ligustilide group.

To clarify whether ligustilide regulated the nuclear translocation of EGR1 to nucleus, Western blot and immunofluorescence were conducted in RAW264.7 cells. Western blot found that after treatment of ligustilide, EGR1 protein expression was significantly decreased in the nucleus but significantly increased in the cytosol (Fig. 5A and B). An immunofluorescence experiment also observed that ligustilide limited the nuclear import of the EGR1 protein (Fig. 5C). To verify whether endogenous EGR1 can affect the anti-inflammation effect of ligustilide, we overexpressed EGR1 using lentiviruses. As demonstrated in Fig. 5D and E, after transfection of lentiviruses, the fluorescence intensity and protein expression level were observably enhanced. Immunofluorescence staining demonstrated that ligustilide disturbed EGR1 nuclear translocation, whereas EGR1 overexpression inhibited this process (Fig. 5F). COX-2 and iNOS are biomarker proteins associated with inflammatory response in macrophage [17,18]. Besides, ligustilide reduced EGR1, iNOS, and COX-2 expressions by Western blot analysis. In contrast, compared with the ligustilide group, EGR1 overexpression significantly elevated iNOS and COX-2 expressions, indicating that ligustilide alleviated macrophage inflammation response through impeding EGR1 activation (Fig. 5G). These results demonstrated that ligustilide could obstruct the nuclear translocation of EGR1, thereby reducing macrophage inflammation response.

### Ligustilide restricted TNF-α production in macrophage by inhibiting the EGR1-ADAM17-TNFα pathway

**Fig. 6.**
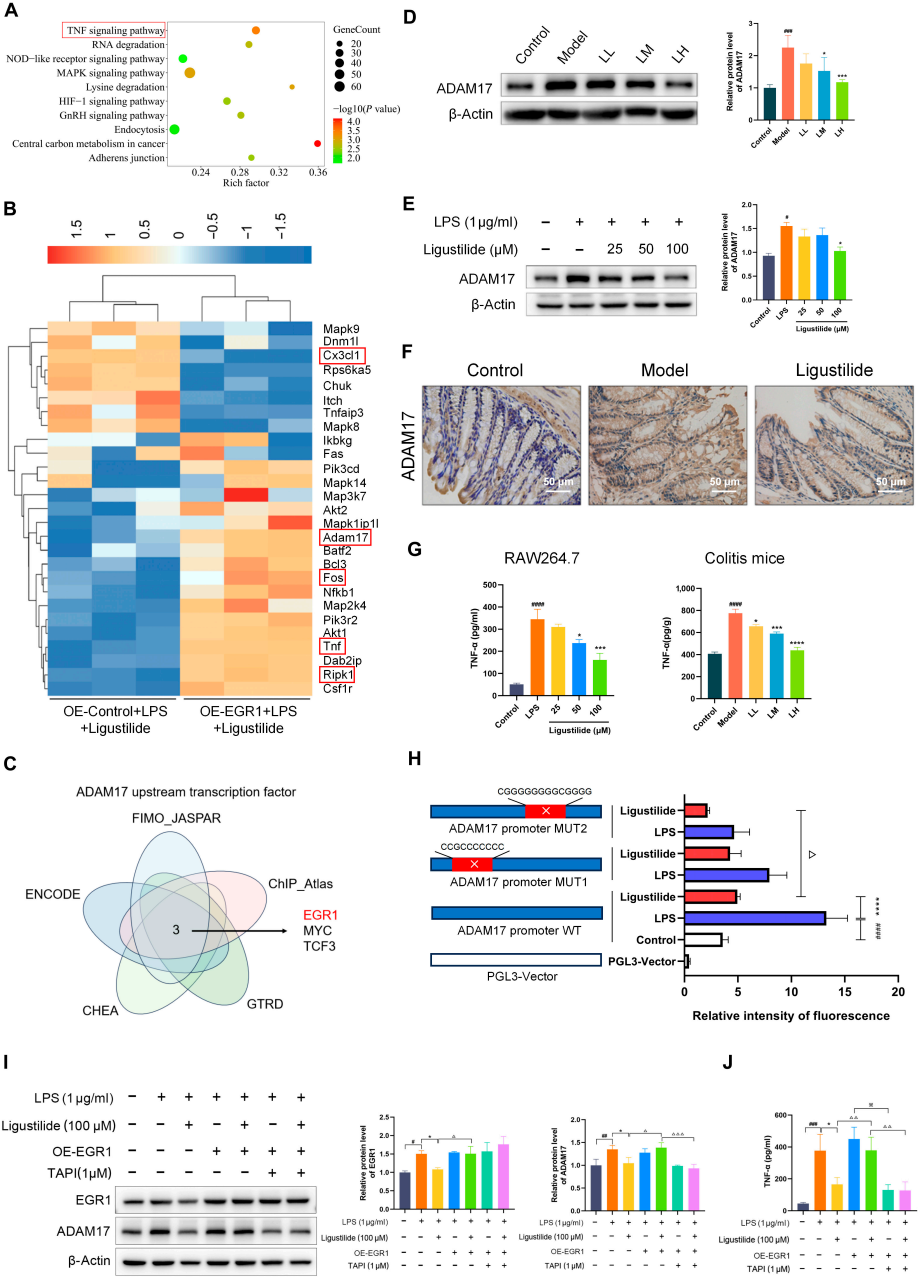
Ligustilide restricted TNF-α production by inhibiting the EGR1-ADAM17 pathway. (A) KEGG analysis. (B) Heatmap of TNF-α signaling-related genes. (C) Bioinformatics analysis of ADAM17 upstream transcription factor based on 5 public databases. (D) Western blot-evaluated expression of ADAM17 in colitis mice. (E) Western blot assay-evaluated expression of ADAM17 in RAW264.7 cell. (F) Immunohistochemistry assay-evaluated expression of ADAM17 in colitis mice. (G) TNF-α levels in RAW264.7 cell and colitis mice. (H) Dual-luciferase reporter gene assay detected the transcriptional activity of ADAM17 on wild-type ADAM17 promoter and mutated promoter in HEK293T cells. (I) Western blot analyzed whether the EGR1-ADAM17 pathway was crucial for EGR1 and ADAM17 expressions. (J) ELISA analyzed whether the EGR1-ADAM17 pathway was crucial for TNF-α production. TAPI-1, TNF-α processing inhibitor-1; OE-EGR1, EGR1 overexpression. LL, low dose of ligustilide; LM, medium dose of ligustilide; LH, high dose of ligustilide. ^#^*P <* 0.05, ^##^*P <* 0.01, ^###^*P <* 0.001, ^####^*P <* 0.0001 vs. control group. ^*^P < 0.05, ^**^P < 0.01, ^***^P < 0.001 vs. model group or LPS group. ^△△^ *P <* 0.01, ^△△△^ *P <* 0.001, ^△△△△^ *P <* 0.001 vs. LPS+Ligustilide+OE-EGR1 group or Ligustilide group, ^※^ *P <* 0.05 vs. LPS+OE-EGR1 group.

To identify the regulatory downstream gene of EGR1 with ligustilide intervention, we used the RNA transcriptomic sequencing in ligustilide-treated OE-Control and OE-EGR1 RAW264.7 cells. As displayed in Fig. 6A, Kyoto Encyclopedia of Genes and Genomes (KEGG) analysis indicated that EGR1 overexpression significantly affected multiple pathways, of which the TNF-α signaling pathway was closely connected with the macrophage inflammatory response in colitis. TNF-α signaling-related genes’ expressions analysis found that OE-EGR1 markedly regulated expression levels of TNF-α signaling-related genes Adam17, Fos, Ripk1, and Cx3cl1 (Fig. 6B). Subsequently, we adopted bioinformatics to analyze the upstream transcription factor of the above genes in 5 public databases and only the upstream transcription factors of ADAM17 contained EGR1, which made it the potential target gene of EGR1 (Fig. 6C and Fig. S5).

Then, differential expression of ADAM17 after ligustilide intervention was analyzed. In DSS-induced colitis mice, ligustilide significantly reduced ADAM17 protein expression by Western blot and immunohistochemistry analysis (Fig. 6D and F). Consistently, ligustilide reduced ADAM17 protein expression in RAW264.7 cells exposed to LPS (Fig. 6E). In addition, ligustilide inhibited the TNF-α signaling pathway, evidenced by a dose-dependent decline of TNF-α content in LPS-stimulated RAW264.7 cells and colitis mice (Fig. 6G). Importantly, dual luciferase reporter gene assay was performed to explicate the essential promoter sequence of ADAM17 that EGR1 binds. Bioinformatics analysis predicted that ADAM17 had 2 binding sequences with EGR1: “CCGCCCCCCC” and “CGGGGGGGGCGGGG”. Two plasmids with mutated sequence were constructed and transfected to HEK293T cells. Interestingly, ligustilide significantly diminished the intensity of fluorescence in HEK293T cells transfected by the WT ADAM17 promoter (*P <* 0.0001). Compared with the ligustilide group, ADAM17 promoter MUT2 mutation enhanced the inhibitory effect of ligustilide on ADAM17 transcription activity (Fig. 6H). Moreover, we carried out rescue experiments to prove that the EGR1-ADAM17 pathway was crucial for TNF-α production. Ligustilide significantly reduced the protein levels of EGR1 and ADAM17, whose effects were neutralized by EGR1 overexpression (Fig. 6I and Fig. S6). Besides, ADAM17 expression and TNF-α content were raised in ligustilide-treated OE-EGR1 cells, while ADAM17 inhibitor TAPI-1 impeded these effects, implying that ADAM17 was crucial downstream mechanism of EGR1 in ligustilide reducing TNF-α production (Fig. 6I and J). These results illustrated that the MUT2 sequence “CGGGGGGGGCGGGG” might be a key binding sequence of the ADAM17 promoter with EGR1. Taken together, ligustilide might reduce macrophage inflammatory response by inhibiting EGR1 transcription and the downstream ADAM17-TNFα pathway.

### Ligustilide improved DSS-induced symptoms and restored intestinal barrier by restraining the EGR1-ADAM17 pathway

**Fig. 7.**
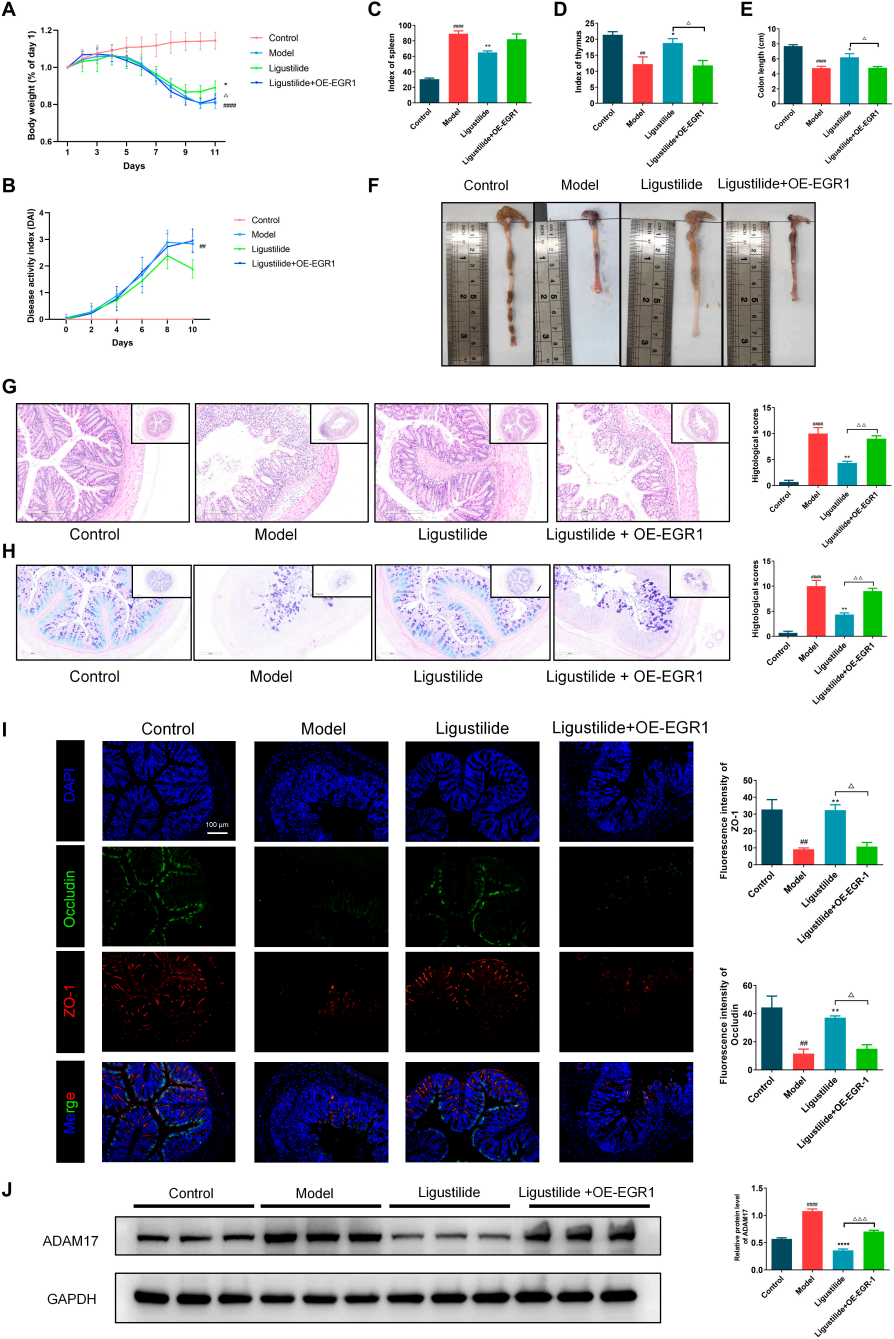
Ligustilide improved DSS-induced symptoms and restored intestinal barrier by restraining the EGR1-ADAM17 pathway. (A) Body weight changes. (B) DAI score changes. (C) Index of spleen. (D) Index of thymus. (E and F) The lengths of colons. (G) H&E staining. (H) AP-PAS staining of goblet cells. (I) Immunofluorescence analysis of ZO-1 and Occludin. (J) Western blot analysis of ADAM17. OE-EGR1, EGR1 overexpression. ^##^*P <* 0.01, ^###^*P <* 0.001, ^####^*P <* 0.0001 vs. control group. ^*^P < 0.05, ^**^P < 0.01, ^****^P < 0.0001 vs. model group. ^△^ *P <* 0.05, ^△△^ *P <* 0.01, ^△△△^ *P <* 0.001, vs. Ligustilide group.

To investigate the potential of EGR1 for ligustilide in improving colitis mice, mice were injected with adeno-associated virus (AAV)-EGR1 to overexpress EGR1 and treated by ligustilide. Compared with the model group, ligustilide delayed DSS-induced weight loss and increased DAI score, and significantly reduced weight and DAI score on the last day, which was abrogated in the context of EGR1 overexpression (Fig. 7A and B). Ligustilide notably increased the thymus index, decreased the spleen index, and lengthened colons. However, these impacts were abolished when EGR1 was overexpressed (Fig. 7C to F). In addition, OE-EGR1 also reversed ligustilide-mediated intestinal barrier restoration, as evidenced by exfoliated epithelial cells, abundant inflammatory cell infiltration, disappearing goblet cells, and an obvious decline in tight junction protein ZO-1 and Occludin expressions (Fig. 7G to I). Furthermore, ligustilide could considerably reduce ADAM17 protein expression, while OE-EGR1 offset this effect with a significant difference (Fig. 7J). Collectively, ligustilide improved DSS-induced UC symptoms and restored intestinal barrier by restraining EGR1 activation.

## Discussion

UC usually occurs in the rectal and colonic mucosa and submucosa. It is commonly accompanied by symptoms such as abdominal pain, diarrhea, weight loss, and bloody and purulent stools [19]. The causes of UC are numerous, including immune disorders, changes in the intestinal microbiota, genetic susceptibility, etc. [20–22]. The clinical drugs for UC exhibited limitations such as high recurrence after drug withdrawal, which drives drug discovery research. Therefore, our study aimed to claim the effect and potential mechanism of ligustilide on UC.

In this study, we administrated DSS-induced colitis mice with ligustilide to assess the anti-colitis activity of ligustilide. In vivo pharmacodynamic results showed that ligustilide significantly attenuated weight loss and anal ulceration; reduced the DAI score; improved organ indices, including reducing the spleen index and increasing the thymus index; and restored the length of the colon in colitis mice. In addition, ligustilide alleviated the infiltration of immune cells in the muscular and plasma layers of the intestinal mucosa and attenuated the damage and abnormality of crypt structures. Therefore, these data proved that ligustilide could improve colitis-associated symptoms.

The intestinal epithelium, located at the junction of the intestinal lumen and the lamina propria, is mainly composed of intestinal epithelial cells, Paneth cells, and goblet cells [23,24]. Under normal circumstances, intestinal epithelial cells isolate pathogens, bacteria, and toxins in the intestinal lumen by forming tight junctions [25,26]. When the intestinal epithelium is damaged, pathogenic microorganisms in the intestinal lumen enter the lamina propria, thereby triggering the infiltration and activation of immune cells. Activated immune cells produce chemokines and pro-inflammatory cytokines such as IL-1β and TNF-α, further exacerbating the injury of the intestinal epithelial barrier and leading to the occurrence of UC, and even the transition to colorectal cancer [27–29]. Our investigation found that ligustilide significantly reduced the bacterial load in mesenteric lymph nodes and decreased the fluorescent area of FD4 in enterocoelia, suggesting that ligustilide could reduce the intestinal permeability of colitis mice. The chemical barrier is composed of mucus secreted by goblet cells and antibacterial peptides secreted by Paneth cells [30]. The mucus layer, as one of the defense lines of the intestinal mucosal barrier, plays an important role in maintaining intestinal homeostasis [31]. Alcain blue and periodic acid-Schiff (AB-PAS) staining results indicated that ligustilide increased the number of goblet cells and protected the mucus layer in colitis mice. Intestinal epithelial cells are interconnected through junction complexes such as tight junctions, adhesion junctions, gap junctions, and desmosomes, maintaining the integrity of the intestinal mucosal barrier. Tight junction proteins are mainly composed of Claudin, Occludin, junction adhesion molecules (such as JAM-A), and ZO proteins [25,32]. It was observed that ligustilide protected the colonic microvilli structure, narrowed the tight junction gap, and enhanced tight junction protein Claudin-1 and Occludin expressions in colitis mice. Thus, our data demonstrated that ligustilide could repair the intestinal mucosal barrier and restore intestinal structural integrity.

Inflammation is the pivotal pathophysiological basis of UC [13–15]. Under an inflammatory microenvironment, monocytes recruited from peripheral blood differentiate into F4/80^+^ macrophages and accumulate in the lamina propria [33,34], which drives inflammatory cascade by producing large amounts of inflammatory cytokines (such as TNF-α, IL-1β, and IL-6) and inflammatory proteins (such as iNOS and COX-2) [35–37]. COX-2, also known as inducible cyclooxygenase, exhibits extremely low activity in normal tissues. While upon inflammatory stimulation, COX-2 is activated rapidly to participate in arachidonic acid metabolism and the release of inflammatory mediators, thereby exacerbating inflammation and tissue damage. Similarly, iNOS activation leads to excessive NO production in response to inflammatory signals. Studies have shown that COX-2 and iNOS mutually activated in inflammatory environments, accelerating the progression of UC [38–42]. As expected, we observed that ligustilide attenuated macrophage infiltration in the colon and reduced IL-1β and TNF-α transcription levels and expressions of iNOS and COX-2 in LPS-induced macrophage and colitis mice, suggesting that the anti-inflammatory mechanism of ligustilide might involve the suppression of macrophage-driven inflammatory responses.

Changes in the thermal stability of proteins are considered essential for protein engagement with ligand [43,44], which is conducive to drug discovery [45]. TPP, which combines the CETSA method and proteomics, is aimed at target protein screening and drug identification [46]. To reveal the target of ligustilide in suppressing macrophage inflammation, TPP was utilized in RAW264.7 cells according to previous researches [47], and EGR1 was preliminarily selected as a potential target owing to its strong stability. EGR1, a key transcription factor, is involved in tissue damage, immune response, and fibrosis processes [5,48,49]. Then, CETSA, DARTS, and SPR experiments proved the solid interaction of ligustilide with EGR1. Additionally, the binding pocket of ligustilide with EGR1 was predicted as His386, and an EGR1 His386 MUT HEK293T cell was constructed by plasmid transfection. The following results showed that EGR1 His386 MUT lowered ligustilide-treated EGR1 stability after being stimulated by gradient temperature and pronase. In sum, ligustilide could interact with EGR1 via binding to His386.

However, whether ligustilide, as a specific binding partner of EGR1, affects the nuclear shift and curbs downstream inflammatory events needs to be examined. Our Western blot and immunofluorescence assays revealed that ligustilide disturbed the nuclear translocation of EGR1 stimulated by LPS, whereas EGR1 overexpression offset this process. Guo et al. [50] evidenced that EGR1 might promote the expression of COX-2 to regulate vascular permeability and angiogenesis during mouse embryo implantation. Harada et al. [51] uncovered that EGR1 knockout dramatically weakened iNOS expression. Consistently, ligustilide exhibited a striking reduction in inflammatory proteins iNOS and COX-2 expressions, which was counteracted by EGR1 overexpression. These findings confirmed that ligustilide mitigated macrophage inflammatory responses through inhibiting the nuclear translocation of EGR1.

To determine the transcriptional mechanism by which ligustilide regulated macrophage inflammation, RNA transcriptomics were conducted and analyzed and TNF-α signaling pathway was significantly enriched by KEGG analysis. Accumulated evidences suggest that TNF-α can induce the activation of necrosis regulatory factors Tnfaip3/A20 and Ripk1, bringing about necrosis of intestinal epithelial cells and further disrupting intestinal barrier function [52,53]. Combined with bioinformatics analysis, we focused on the TNF-α signaling-related gene ADAM17. ADAM family proteins are membrane-anchored albumin, which perform functions such as hydrolysis and cutting of cell surface protein, signal transduction, and cell adhesion, engaging in neurogenesis, immune regulation, and inflammatory diseases [6]. ADAM17 can cut the TNF-α precursors, followed by processing and releasing active TNF-α. Studies have shown that UC mice with a high expression of ADAM17 have a significantly increased susceptibility to inflammation, severe intestinal mucosal barrier damage, and elevated expression of pro-inflammatory factors such as TNF-α, and histopathological results showed tissue destruction, loss of barrier integrity, and infiltration of immune cells [54,55].

Colón et al. [56] demonstrated that an increase in activity and expression of ADAM17 led to TNF-α release and iNOS activation, aggravating 2,4,6-trinitro-benzene sulfonic acid (TNBS)-induced colitis. Likewise, we observed that ligustilide obviously weakened ADAM17 expression in colitis mice and LPS-activated macrophage. We further examined ADAM17 and TNF-α level via a rescue experiment (EGR1 overexpression and ADAM17 knockdown). Interestingly, EGR1 overexpression elevated ADAM17 expression and TNF-α production, while ADAM17 inhibitor TAPI-1 overturned these effects. Finally, a dual-luciferase reporter gene assay verified that mutation of the ADAM17 promoter sequence “CGGGGGGGGCGGGG” intensified the inhibitory effect of ligustilide on the transcription activity of ADAM17, implying that ligustilide hindered the combination between EGR1 and the ADAM17 promoter sequence “CGGGGGGGGCGGGG”. These discoveries illustrated that ligustilide inhibited TNF-α release by hindering the interaction of EGR1 and ADAM17 promoter and ADAM17 transcription.

Finally, we utilized AAV-EGR1 to overexpress EGR1 in vivo and evaluate the potential role of EGR1 as an essential target for ligustilide in colitis mice. Ligustilide treatment reduced DSS-triggered weight loss, increased DAI scores, reduced spleen enlargement and thymic atrophy, increased colon length, restored colonic pathological structure, increased expressions of ZO-1 and Occludin, and decreased EGR1 downstream target protein ADAM17 expression. However, these effects were abolished with EGR1 overexpression. Taken together, EGR1 downregulation contributes to the inflammatory balance and gut barrier protective effects of ligustilide in colitis mice.

**Fig. 8.**
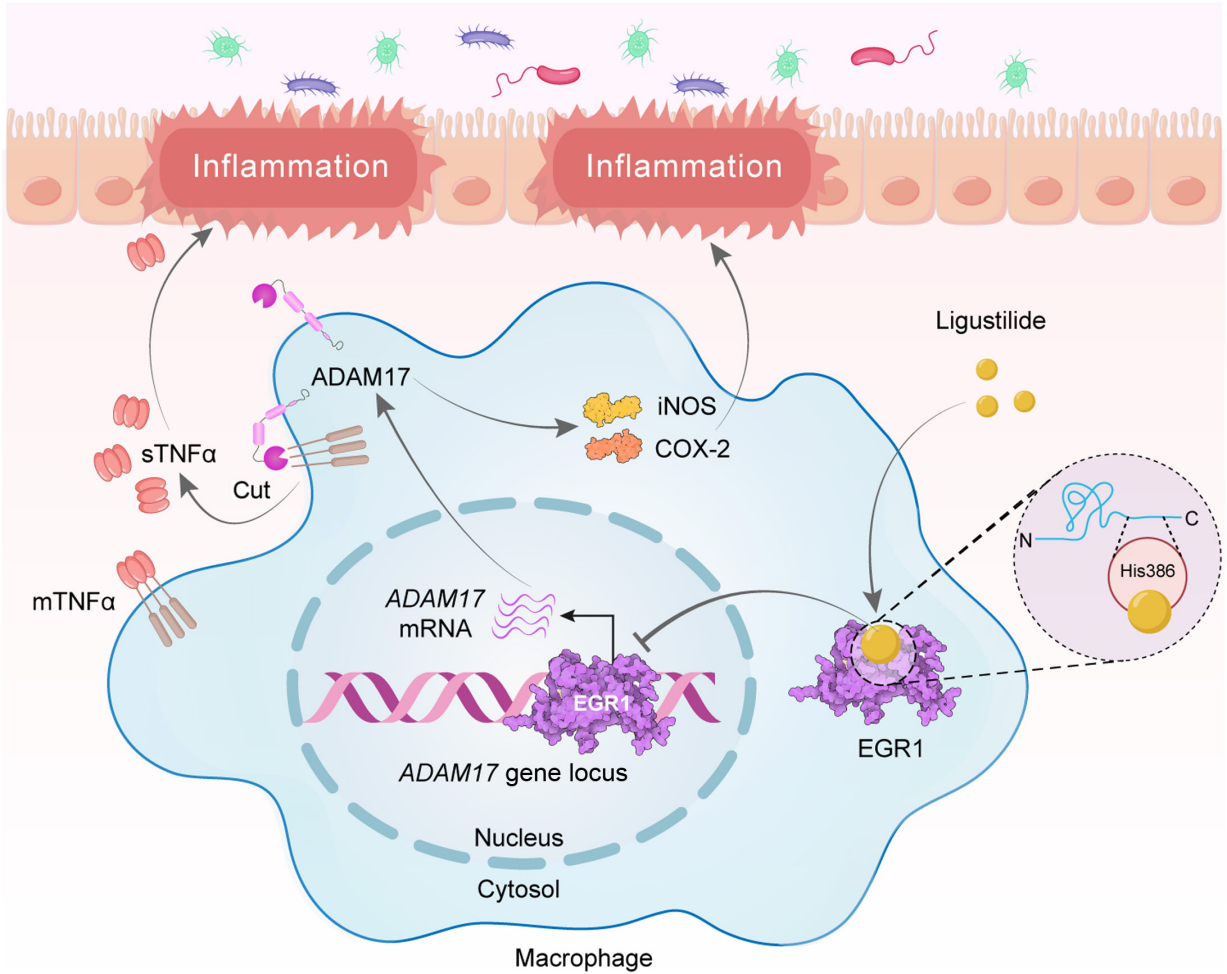
Mechanism of ligustilide on relieving colitis by inhibiting the EGR1-ADAM17-TNFα signaling pathway.

In summary, our study demonstrates that ligustilide targets EGR1 to inhibit the EGR1-ADAM17-TNF-α pathway, thus alleviating macrophage-mediated intestinal inflammation and restoring gut barrier (Fig. 8).

## Materials and Methods

### Reagents

Ligustilide (>98% purity, WKQ-0004558) was purchased from Sichuan Weikeqi Biological Technology Co., Ltd. (Sichuan, China). Sulfasalazine (599-79-1) was purchased from MedChemexpress Biotech Ltd. (NJ, USA). DSS (D2910011) was purchased from Yeasen Biotech Co., Ltd. (Shanghai, China). 10% ExpressCast PAGE Gel Preparation kit (P2012) was purchased from New Cell & Molecular Biotech Co., Ltd. (Suzhou, China). IL-1β ELISA (enzyme-linked immunosorbent assay) Kit (MM-0040M1) and IL-6 ELISA Kit (MM-0163M1) were purchased from Jiangsu Meimian Industrial Co., Ltd. (Jiangsu, China). Mouse TNF-α ELISA Kit (RX202412M) was purchased from Quanzhou Ruixin Biotechnology Co., Ltd. (Quanzhou, China). Occludin (27260-1-AP), Claudin-1 (13050-1-AP), iNOS (18985-1-AP), and ADAM17 (29948-1-AP) antibodies were purchased from Proteintech Group, Inc. (Wuhan, China). ZO-1 (ab96587), E-cadherin (ab76319), and EGR1 (ab300449) antibodies were purchased from Abcam (Cambridge, UK). COX-2 (AF7003) antibody was purchased from Affinity (Melbourne, USA). AAV-EGR1 was purchased from Shanghai Genechem Co., Ltd (Shanghai, China).

### Establishment of the UC mouse model and administration of drugs

C57BL/6J male mice (6 to 8 weeks, 20 to 22 g) were used, with production license number SCXK (Guangdong) 2021-0057 from Guangdong Zhiyuan Biomedical Technology Co., Ltd. The experimental animals were kept in an SPF laboratory animal environment. Animal experimental studies were approved by the Animal Ethics Committee of the School of Chinese Medicine, Guangzhou University of Chinese Medicine [license number: SYXK (Yue) 2024-0202].

All mice were randomly divided into 6 groups: control group, model group, low dose of ligustilide group (LL, 12.5 mg/kg), medium dose of ligustilide group (LM, 25 mg/kg), high dose of ligustilide group (LH, 50 mg/kg), and sulfasalazine group (SASP, 200 mg/kg). Mice except those from the control group received DSS for 7 days, mice except those from the control group and the model group were administrated with ligustilide and SASP for 10 days. During the experiment, the body weight, fecal traits, and hematochezia of mice were recorded daily, and then the DAI scores were calculated following the scoring criteria [57]. On the 11th day, mice were euthanized, and peripheral blood was collected for blood cell analysis. Spleen and thymus were obtained for weighing. Colons were collected for measurement and photographing.

### The index of spleen and thymus

The spleen and thymus of mice were collected for organ index calculation abided by the formula, which was as follows [58].

$$\begin{array}{c} \text{Index of thymus}=\\ \text{wet weight of thymus}\;(\text{mg})\times 10/\text{body weight}\;(g) \end{array}$$ (1)

$$\begin{array}{c} \text{Index of spleen}=\\ \text{wet weight of spleen}\;(\text{mg})\times 10/\text{body weight}\;(g) \end{array}$$ (2)

### Histopathological analysis

The colon, about 1 cm long near the upper rectum, was clipped, soaked, and fixed in 4% paraformaldehyde for 48 h for subsequent pathological tissue staining. After dehydration and embedding, paraffin sections with 4 μm thickness were made and stained with hematoxylin and eosin (H&E) and AB-PAS. Images were observed and collected using a microscope, and histopathological scores on the collected images were determined according to the histopathological scoring criteria [59].

### Cell culture and experiment

EGR1 overexpression RAW264.7 cells was established by Xi’an Setobotai Biotechnology Co., Ltd. (Xi’an, China) and identified by short tandem repeat (STR). HEK293T cells, OE-EGR1 RAW264.7 cells, and RAW264.7 cells were cultured in a 37 °C, 5% CO_2_ incubator.

Cells were pretreated with ligustilide with different concentrations (25, 50, and 100 μM) for 2 h and stimulated by LPS (1 μg/mL) for 6 h.

### Immunofluorescence

The paraffin section of the colon was dewaxed with xylene and ethanol, microwaved for antigen retrieval, and blocked with 3% bovine serum albumin. Then, the paraffin section was incubated with primary antibody at 4 °C overnight. The next day, the primary antibody was washed with phosphate-buffered saline (PBS) and incubated with the secondary antibody for 1 h. Finally, the nucleus was re-stained with 4′,6-diamidino-2-phenylindole (DAPI) solution. Fluorescence images were collected by fluorescence microscopy.

### Immunohistochemistry

The fixed colon tissue was embedded in paraffin wax and cut into sections with a thickness of 4 μm. The tissue sections were incubated with primary antibody ADAM17 at 4 °C overnight. The next day, after washing 3 times with PBS, the secondary antibody was incubated at 37 °C for 1 h. Finally, the sections were observed under an optical microscope.

### Quantitative real-time polymerase chain reaction

Total RNA of cells was extracted, reverse transcribed, and amplified following the protocols of the manufacturer. The primer sequences are listed in Table S1.

### Small-animal imaging

After fasting for 12 h, all mice were administered with 50 mg/kg 4 kDa fluorescein isothiocyanate dextran (FD4) intragastrically. After 4 h, the distribution of FD4 in the gastrointestinal tracts was observed by the small-animal imaging system.

### Transmission electron microscopy

The colon tissue was gently rinsed on ice with pre-cooled PBS, then quickly put into the electron microscope fixation solution, and the tissue was cut into 1-mm^3^ tissue blocks with a blade and fixed for 4 h, then rinsed with PBS, fixed with 1% osmic acid solution for 3 h, and dehydrated with 30%, 50%, 70%, 80%, 95%, and 100% acetone solution. After dehydration, it was soaked and embedded with acetone-Epon812 resin. Finally, the frozen slices were cut into 60-nm ultrathin slices by a freezing microtome. The ultrastructure of the tight epithelial tight junction in colon tissue was observed by TEM, and the images were collected for analysis.

### Bacterial culture

Spleen and MLNs of mice were separated and collected, placed in 0.9% normal saline, and ground at 60 Hz for 2 min by a homogenizer. Then, they were centrifuged at 4 °C, 3,000 rpm for 3 min and the supernatant was collected. Subsequently, 20 μl of the supernatant was added to Luria–Bertani agar, uniformly coated with disposable coating rods, and cultured at 37 °C for 12 h. Finally, the bacterial colonies were photographed and calculated.

### Western blot

Proteins of cells or colon tissues were denatured and separated by sodium dodecyl sulfate - polyacrylamide gel electrophoresis (SDS-PAGE) gel electrophoresis. Then, proteins were transferred from a polyacrylamide gel to a polyvinylidene fluoride (PVDF) membrane at 300 mA for 90 min. After blocking with 5% non-fat powdered milk powder for 2 h, the PVDF membrane was incubated with the primary antibody overnight. The next day, the PVDF membrane was incubated with goat anti-mouse IgG/HRP for 1.5 h at room temperature. Following 3 times washing by PBS, the PVDF membrane was imaged using a chemiluminescence imager, and gray scale analysis was performed on the bands using ImageJ.

### Enzyme-linked immunosorbent assay

The contents of IL-1β, IL-6, and TNF-α were detected by using an ELISA kit according to protocols.

### Molecular docking

The 3-dimensional structure of ligustilide was derived from the PubChem database. The EGR1 protein structures were derived from the Protein Data Bank database (https://www.rcsb.org/). AutoDock Tools 1.5.7 was used to dock the compound and the target protein, and PyMOL 2.1 was used to visualize the docking results.

### Thermal proteome profiling

RAW264.7 cells with 90% to 100% density were collected and lysed with lysis buffer mixed with 1 mM phenylmethylsulfonyl fluoride for protein extraction. Then, protein samples were divided into the DMSO group and the ligustilide group, of which proteins in the ligustilide group were treated with ligustilide, while proteins in the DMSO group were treated with DMSO for 1 h at room temperature. After incubation, all protein samples were heated at 60 °C for 3 min. Finally, proteins were identified by proteomics analysis performed by Shanghai OE Biotech Co., Ltd.

### Drug affinity responsive target stability

When the density of cells reached 90% to 100%, PBS was added and washed 2 times. Then, the cell lysis buffer was added, and ultrasonic lysis was performed on ice for 15 min. The mixture was centrifuged at 4°C, 12,000 *g* for 15 min, and the supernatants were collected as protein samples. Subsequently, protein samples were mixed with TNC buffer and divided into 5 groups. DMSO or ligustilide (25, 50, and 100 μM) was added and incubated respectively at room temperature for 1 h. After incubation, protein samples were decomposed by pronase for 30 min at 37 °C. Following decomposing, protein samples were boiled for denaturation. Finally, we utilized the protein samples for Western blot.

### Cellular thermal shift assay

Specifically, when the density of cells reached 90% to 100%, proteins of cells were extracted by cell lysis buffer. Then, ligustilide (100 μM) was added and incubated for 1 h. Following incubation, protein solution was aliquoted into 8 EP tubes and heated at the specified gradient temperatures (43, 46, 49, 52, 55, 58, 61, and 64 °C) for 3 min. Subsequently, the solution was centrifuged at 20,000 *g* for 20 min and the supernatants were collected. 5× loading buffer was added to the supernatants and boiled for 5 min. Finally, the denatured protein samples were used to perform Western blotting.

### RNA-sequencing

Total RNA of RAW264.7 cells was extracted using the Universal RNA Extraction CZ Kit. RNA purity was analyzed using Qubit 4.0 and the quality was assessed by electrophoresis. Enrichment of mRNA, construction of library, sequencing, and bioinformatics analysis were conducted by Shanghai Xu Ran Biotechnology Co., Ltd.

The raw data were handled by Skewer v0.2.2. The differentially expressed genes (DEGs) were analyzed by DESeq2 (v1.16.1). The thresholds for DEGs are *P* < 0.05 and fold change ≥ 2. Finally, function and signaling pathway enrichment analysis were performed by the TopGO and KEGG database.

### Molecular dynamics simulation

Molecular dynamics simulation was conducted using Amber 24. Solvation was carried out using the TIP3P water model, and counterions were added to neutralize the system. Once the system energy is minimized, the system is heated from 0 to 310.15 K within 500 ps. System constraints were imposed in the normative set ensemble, and then system pre-balancing was carried out at 310.15 K. Finally, a 50-ns molecular simulation was conducted. The dynamic results were analyzed using AmberTools23 [60,61].

### Luciferase reporter assay

PGL3-BASIC, PGL3-ADAM17-WT, PGL3-ADAM17-MUT1, PGL3-ADAM17-MUT2, pCMV-MCS-3 × Flag-EGR1, and PRL-TK plasmids were constructed by Mailgene Biosciences Co., Ltd. and transfected to HEK293T cells for 6 h when the cellular density reached 90%. After transfection, transfection solution was discarded and ligustilide (100 μM) was added for 24 h. Then, all cells were collected to observe luciferase signals using a Dual Luciferase Reporter Assay Kit.

### SPR assay

The interaction of EGR1 with ligustilide was analyzed by SPR using Biacore T200. The optimal pH of recombinant protein EGR1 was 4.5 by pH screening. Then, recombinant protein EGR1 was immobilized on a CM5 chip via amine coupling. Ligustilide was diluted to different concentrations (ranged from 0.82 to 200 μM) and flowed through CM5 chip surface to bind to EGR1. Finally, the affinity constant *K*_D_ was calculated to evaluate the affinity of EGR1 with ligustilide.

### Statistical analysis

Dunnett’s T3 tests were used for the statistical significance between groups when the variance was heterogeneous. Tukey’s tests were used for the statistical significance between groups when the variance was homogeneous. *P* < 0.05 was considered statistically significant.

## Data Availability

All data of this study are available from the corresponding authors upon reasonable request.
