# Supplementary material for: Ligustilide Suppresses Macrophage-Mediated Intestinal Inflammation and Restores Gut Barrier via EGR1-ADAM17-TNF-α Pathway in Colitis Mice
Source: Research (Wash D C). 2025 Sep 2;8:0864. doi: 10.34133/research.0864 (PMC12404647; doi:10.34133/research.0864)
Supplement: Supplementary 1 — Figs. S1 to S6 Table S1 [file research.0864.f1.docx]

**Ligustilide Suppresses Macrophage-mediated Intestinal inflammation and Restores Gut Barrier via EGR1-ADAM17-TNF-α pathway in Colitis Mice**

Yanyang Li ^1,†^, Yequn Wu ^1,†^, Jing Liang ^1^, Peiqi Chen ^1^, Shihua Xu ^1^, Yumei Wang ^1^, Zhi Jiang ^2^, Xudong Zhu ^3^, Chaozhan Lin ^1,*^, Yang Yu ^1,*^, Hailin Tang ^4,*^

^1^ School of Pharmaceutical Sciences, Guangzhou University of Chinese Medicine, Guangzhou, 510006, China.

^2^ Department of Perioperative Research Centre of Chinese Medicine, the Second Affiliated Hospital of Guangzhou University of Chinese Medicine, Guangzhou, 510120, China.

^3^ Markey Cancer Center, University of Kentucky, Lexington, KY, 40536, USA

^4^ State Key Laboratory of Oncology in South China, Guangdong Provincial Clinical Research Center for Cancer, Sun Yat-sen University Cancer Center, Guangzhou 510060, China.

† These authors contributed equally to this work.

* Address correspondence to: [tanghl@sysucc.org.cn](mailto:tanghl@sysucc.org.cn) (H.T.); [yuyang@gzucm.edu.cn](mailto:yuyang@gzucm.edu.cn) (Y.Y.); [linchaozhan@gzucm.edu.cn](mailto:linchaozhan@gzucm.edu.cn) (C.L.)

**Supplementary methods**

**Cell viability assay using CCK8**

The cell concentration of RAW264.7 was adjusted and 1×10^4^ cells/well were seeded into a 96-well plate. When the density of cell ranges to 80%, ligustilide with 3.125, 6.25, 12.5, 25, 50, 100, and 200 μM were intervented for 8 hours. Then, 10 μL CCK8 was added to each well in the dark and incubated in the incubator for 90 minutes. Finally, the OD values of each well were detected at 450 nm wavelength for calculation of cell viability.

**
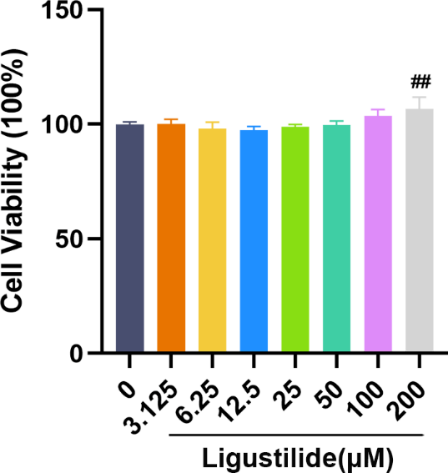
Supplementary figures**

**
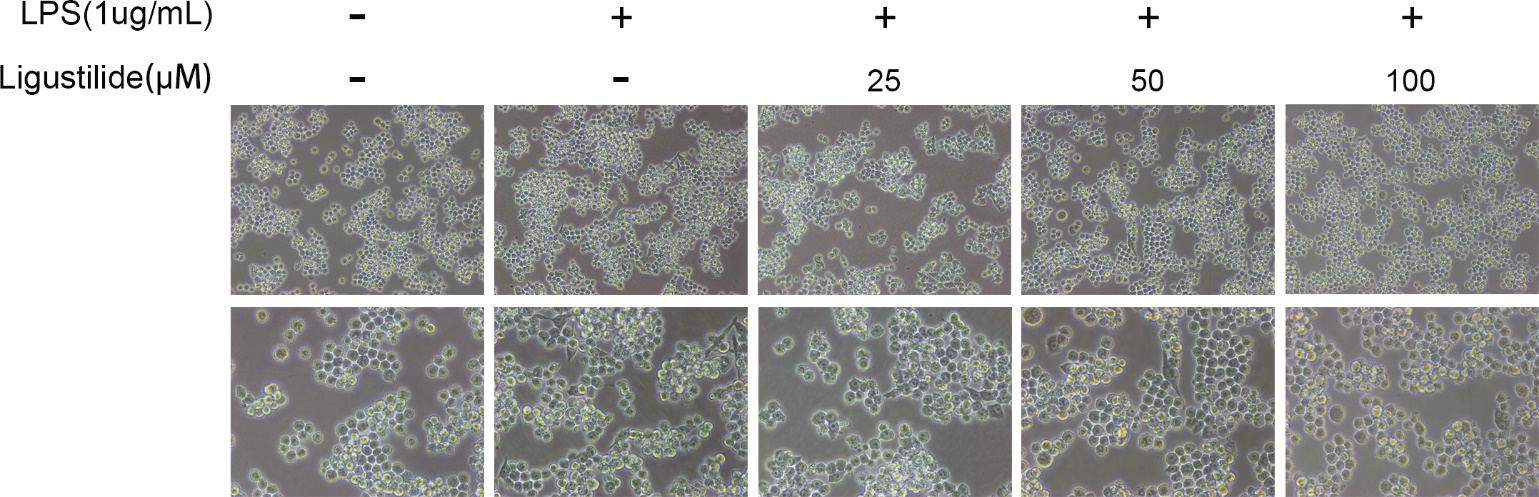
Figure S1.** Cell viability of RAW264.7 cells intervented by ligustilide with gradient concentration using CCK8 detection. *^##^ P* < 0.01 vs. 0 group.

**
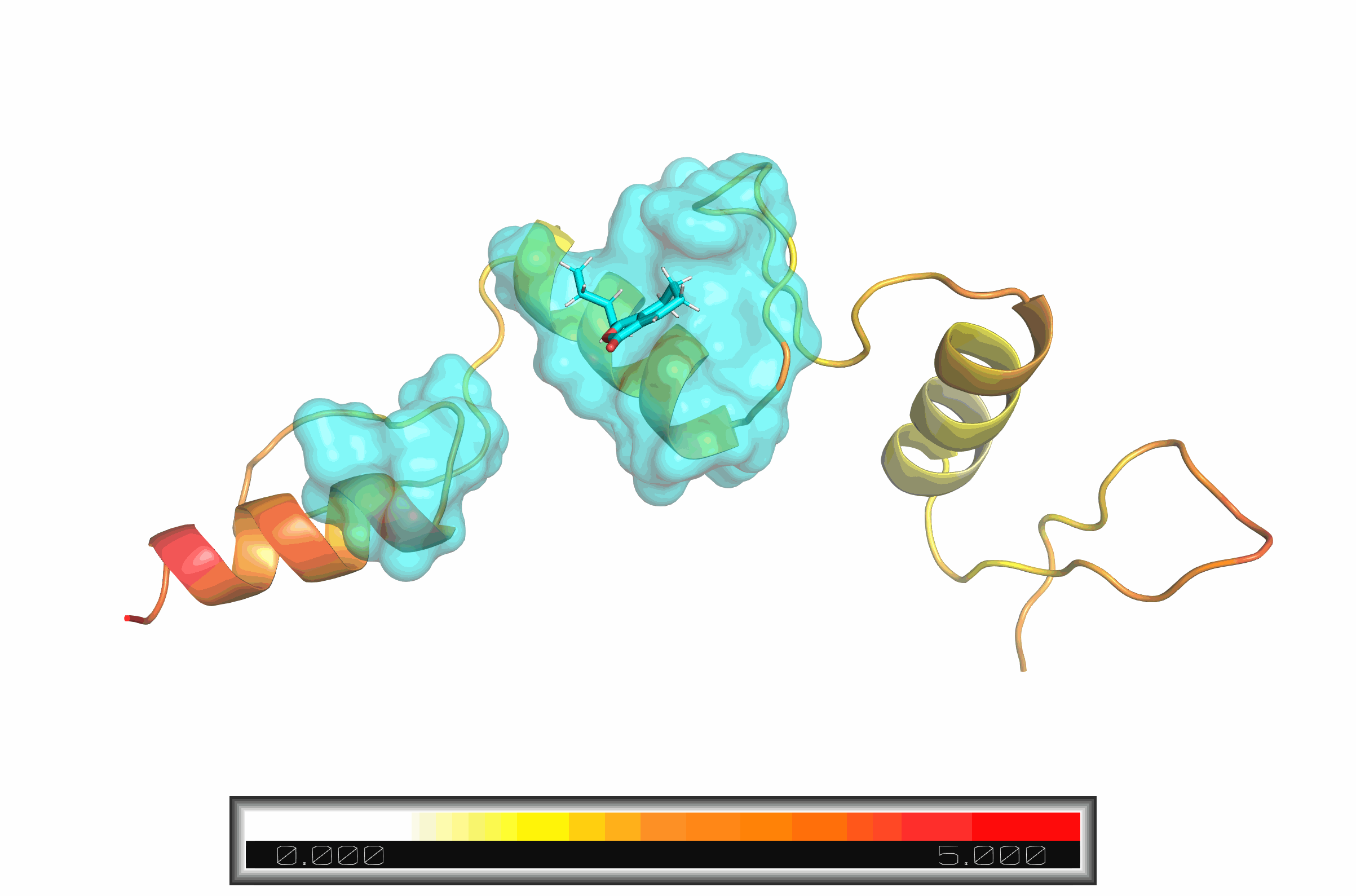
Figure S2.** The effect of ligustilide on the morphology of LPS-induced RAW264.7 cells.

**Figure S3.** Visualization analysis of the binding trajectory of Ligustilide and EGR1.

**
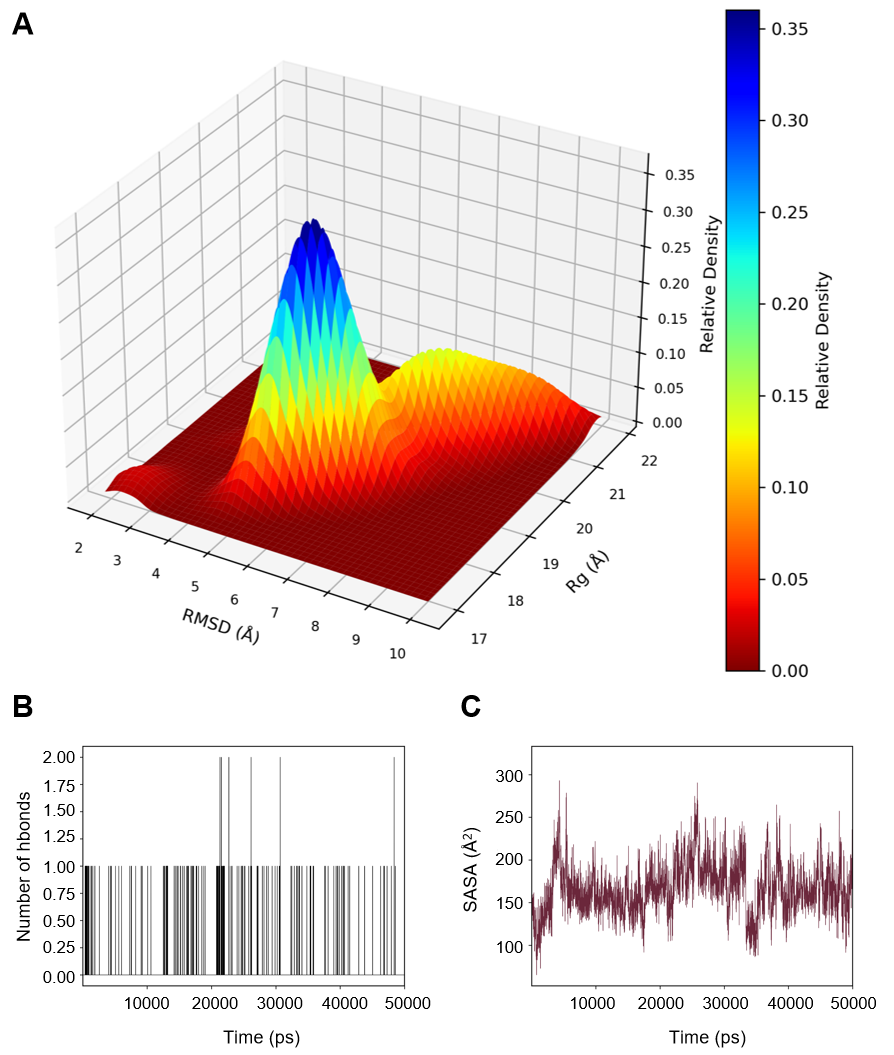
Figure S4.** Molecular dynamics simulation of Ligustilide-EGR1 complex. A) 3D Gibbs free energy landscape of the ligustilide-EGR1 complex. G) solvent-accessible surface area (SASA) of ligand ligustilide and EGR1 protein. H) The number of hydrogen bonds between CHPF2 and ponicidin.

**
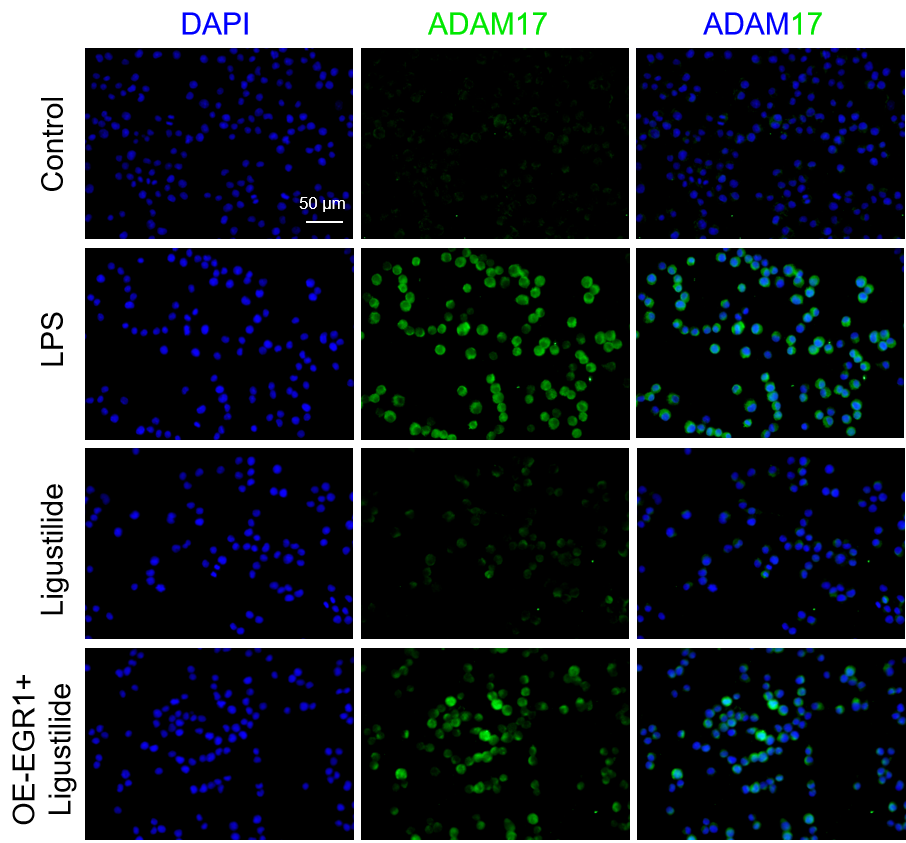
****Figure S5. Bioinformatics predicts the upstream transcription factors of RIPK1, FOS, and CXCL1.
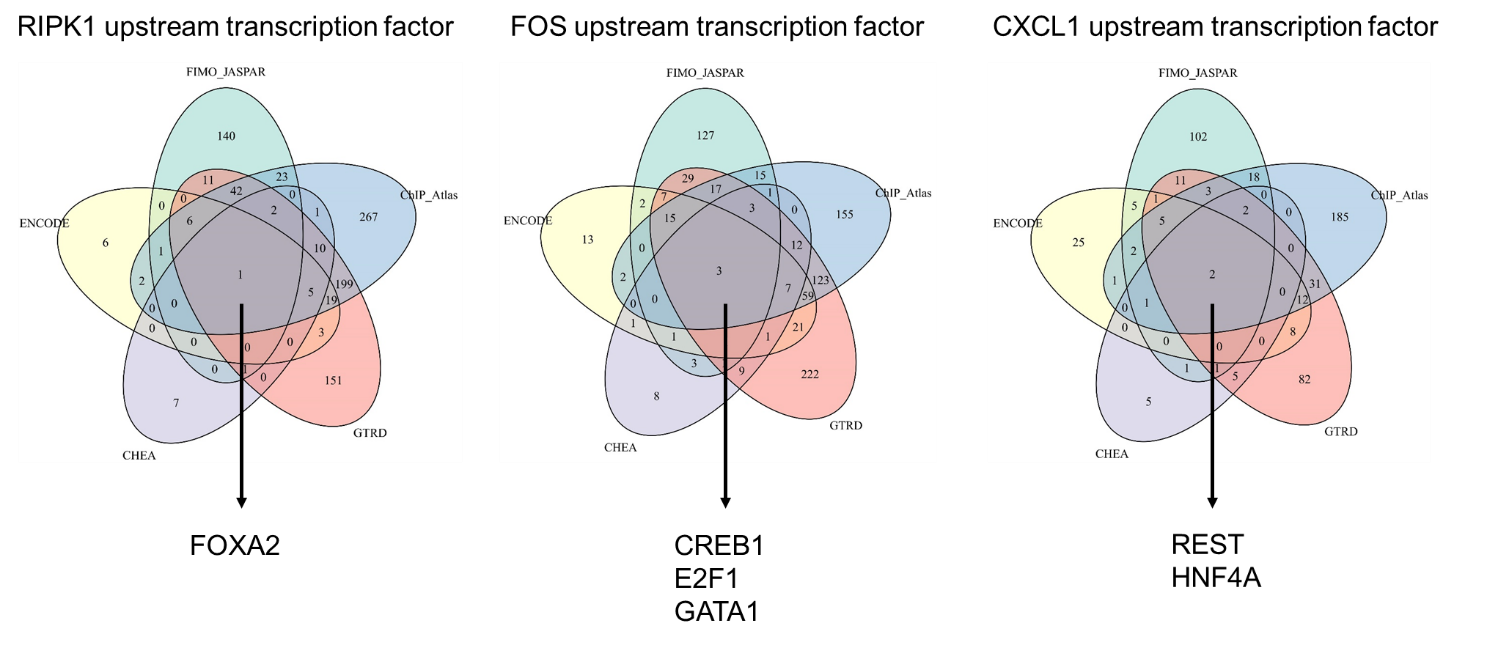
**

**Figure S6. EGR1 is essential in ligustilide inhibiting ADAM17 expression.**

**Supplementary table**

**Table S1. Sequence of primers for qRT-PCR**

| Name | Forward | Reverse |
| --- | --- | --- |
| IL-1β | GAAATGCCACCTTTTGACAGTG | GAAATGCCACCTTTTGACAGTG |
| TNF-α | CAGGCGGTGCCTATGTCTC | CGATCACCCCGAAGTTCAGTAG |
